# Abnormally Expressed Ferroptosis-Associated FANCD2 in Mediating the Temozolomide Resistance and Immune Response in Glioblastoma

**DOI:** 10.3389/fphar.2022.921963

**Published:** 2022-06-08

**Authors:** Liying Song, Jiali Wu, Hua Fu, Cuifang Wu, Xiaopei Tong, Mingyu Zhang

**Affiliations:** ^1^ Department of Pharmacy, The Third Xiangya Hospital, Central South University, Changsha, China; ^2^ Department of Otolaryngology, Hunan Want Want Hospital, Changsha, China; ^3^ Department of Pathology, The Third Xiangya Hospital, Central South University, Changsha, China; ^4^ Department of Neurosurgery, Xiangya Hospital, Central South University, Changsha, China; ^5^ National Clinical Research Center for Geriatric Disorders, Xiangya Hospital, Central South University, Changsha, China

**Keywords:** FANCD2, ferroptosis, temozolomide resistance, glioblastoma, immune microenvironment

## Abstract

Ferroptosis-related genes (FRGs) have been identified as potential targets involved in oncogenesis and cancer therapeutic response. Nevertheless, the specific roles and underlying mechanisms of FRGs in GBM and temozolomide (TMZ) resistance remain unclear. Through comprehensive bioinformatics, we found that ferroptosis-related Fanconi anemia complementation group D2 (FANCD2) was significantly up-regulated in GBM tissues, and the high expression level of FANCD2 was related to the poor prognosis in primary and recurrent GBM patients. Furthermore, FANCD2 could promote TMZ resistance by attenuating ferroptosis in GBM cells. Knockdown of FANCD2 could increase reactive oxygen species (ROS) levels and inhibit cell survival. The two characteristics were associated with ferroptosis in TMZ-resistant GBM cells T98G-R and U118-R. The Kyoto Encyclopedia of Genes and Genomes (KEGG) pathway analysis indicated that aberrantly expressed FANCD2 was potentially linked with several cancer-associated signaling pathways, including chromosome segregation, DNA replication, and cell cycle transition. In addition, we demonstrated that FANCD2 expression was positively correlated with several tumor-infiltrating lymphocytes (TILs) and multiple immune-associated signatures in GBM. Therefore, up-regulated FANCD2 could protect GBM cells from ferroptosis and promote TMZ resistance. FANCD2 may be a novel therapeutic target in GBM.

## Introduction

Glioblastoma (GBM) is one of the most aggressive and lethal tumors. The GBM patients have high recurrence with a median recurrent time of 6.9 months. In the past 30 years, few clinical outcomes of GBM have been improved. For GBM patients, the median survival time is 14.4 months, and the 5-year survival rate is 5.6% (Louis et al., 2021). Nowadays, the treatment strategies for GBM mainly include surgical resection, radiotherapy, and chemotherapy. The temozolomide (TMZ)-based chemotherapy represents a promising management paradigm for treating GBM patients. However, there are still great challenges to improving therapeutic effects due to the high rate of post-therapeutic TMZ resistance and poor prognosis (Yan et al., 2016; Hu et al., 2021). Emerging targeted therapies, such as immunotherapy, are recommended to treat GBM patients in clinical practice (Yan et al., 2020). Adding DCVax-L autologous dendritic cell vaccine into standard therapy can extend the survival time in newly-diagnosed GBM patients (Liau et al., 2018). Nevertheless, only a small minority of GBM patients can benefit from immunotherapy because of the heterogeneity of malignancy (Zhang et al., 2020). Consequently, exploring predictive biomarkers and potential therapeutic targets is imperative to improve outcomes for GBM patients.

Ferroptosis is an iron-dependent regulated cell death characterized by alterations of cellular iron homeostasis and excessive accumulation of lipid peroxides (Yan et al., 2021). Nowadays, increasing studies have identified that ferroptosis-associated modulators are implicated in tumor development and treatment. For instance, as a ferroptosis inducer, sorafenib exerts promising anti-tumor activities by inhibiting the P62-Keap1-Nrf2 signaling pathway *in vitro* and *in vivo* (Sun et al., 2016). In GBM, the inhibition of ferroptosis always contributes to the malignant characteristics and is closely related to poor prognosis. Activating ferroptosis can attenuate the immunosuppressive microenvironment and ameliorate the efficacy of immune checkpoint blockade (Liu et al., 2022). However, the specific effects and potential mechanisms of ferroptosis in GBM pathogenesis and therapeutic response remain unclear.

Fanconi anemia-associated gene, Fanconi anemia complementation group D2 (FANCD2), plays a key role in DNA inter-strand crosslinks repair upon stress. The FANCD2 inhibits ferroptosis-mediated damage by blocking iron accumulation and lipid peroxidation in bone marrow stromal cells (Song et al., 2016). In esophageal squamous cell carcinoma, depletion of FANCD2 protein suppresses cancer proliferation and metastasis by inhibiting cyclin-CDK and ATR/ATM signaling (Lei et al., 2020). Emerging evidence has indicated that FANCD2 inhibition can sensitize cancer cells to therapeutic agents. For instance, FANCD2 RNAi can enhance the sensitivity of lung cancer cell lines to cisplatin and oxaliplatin (Kachnic et al., 2010). The FANCD2 overexpression promotes the resistance to PARP inhibitors in BRCA1/2 deficient cancer cells (Kais et al., 2016). However, the roles of FANCD2 in prognosis, immune infiltration, and chemoresistance in GBM remain unclear.

In this study, we explored the detailed effects of FANCD2 in malignant biological behaviors of GBM. Subsequently, the relationship between FANCD2 expression and tumor-infiltrating lymphocytes (TILs) in GBM was also analyzed. We revealed that as a ferroptosis-related gene (FRG), FANCD2 influenced the progression and prognosis of GBM patients. Furthermore, silencing FANCD2 enhanced the TMZ sensitivity in GBM cells T98G-R and U118-R. These results have indicated that FANCD2 can be a biomarker for GBM prognosis and TMZ sensitivity. Furthermore, FANCD2 can regulate the immune response in GBM.

## Materials and Methods

### Data Collection

We searched several GBM datasets from Gene Expression Omnibus (GEO) database using the followed inclusion criteria: (Louis et al., 2021): Cancer Type: GBM. (Yan et al., 2016). Analysis Type: cancer tissue vs normal brain tissue. Three GBM datasets from GEO were identified, such as GSE2223, GSE4290 and GSE15824 ((Bredel et al., 2005; Sun et al., 2006; Grzmil et al., 2011)). Then, the differential expressed genes (DEGs) between GBM tissues and normal brain tissues were screened out using the GEO2R tool (Table 1). The cut-off value was as follows: *p*-value *<* 0.01 and | logFC| *>* 1. Next, we searched and downloaded the FRGs in GBM from the FerrDb database (Zhou and Bao, 2020), and identified the overlapping genes among DEGs and FRGs through Venn analysis provided by Omicstudio (https://www.omicstudio.cn/tool/6).

**TABLE 1:** The primary characteristics of three GEO datasets on gene expression profiling via microarray.

GEO Datasets	Platform	Sample Size		Up-Regulated Genes	Down-Regulated Genes	Co-DEGs	Refs
**—**	**—**	**Cancer**	**Normal**	**—**	**—**	**3 up-regulated genes**	**—**
GSE2223	GPL1833	50	4	650	891		16204036
GSE4290	GPL570	30	3	1873	1969	2 down-regulated genes	16616334
GSE15824	GPL570	157	23	1770	1708		21406405

### Bioinformatics Analyses

The prognostic values of overlapping genes in GBM patients were performed in the Chinese Glioma Genome Atlas (CGGA) (Zhao et al., 2021). The expression levels of FANCD2 in GBM were analyzed in the three GEO datasets and the TCGA_GTEx-GBMLGG dataset. Using the Xiantao bioinformatics toolbox (https://www.xiantao.love/products), we also explored the relationship between FANCD2 expression and patients’ clinical parameters, such as age, WHO grade, IDH status, and 1p/19q codeletion. Next, we utilized LinkedOmics (Vasaikar et al., 2019) to perform the enrichment analysis of gene ontology biological process (GO-BP) and Kyoto Encyclopedia of Genes and Genomes (KEGG) pathways. The correlation of FANCD2 expression with several immune features in GBM patients was assessed using the TISIDB platform (Ru et al., 2019).

### Cell Transfection

According to previous studies, two TMZ-resistant GBM cell lines (T98G-R and U118-R) were established and cultured (Yan et al., 2019; Lu et al., 2022). Small interfering RNAs (siRNAs) targeting FANCD2 were purchased from Genepharma (Suzhou, China). The sequences were as follows: siFANCD2-1 GTC​CTA​TAT​TCC​TAA​CTG​A and siFANCD2-2 GAC​CCA​AAC​TTC​CTA​TTG​A. Transfection was performed using Lipofectamine 3,000 reagent (Invitrogen, United States) according to the manufacturer’s protocol. After the indicated incubation times, the cells were harvested and subjected to protein extraction or other cellular experiments.

### Western Blot

Protein extraction was performed according to a previous study (Zhu et al., 2021). Protein samples were separated by SDS-PAGE, transferred to polyvinylidene difluoride membrane, and hybridized with the antibodies specific to FANCD2 (28619-1-AP, Proteintech) and β-actin (8432, Santa Cruz). Protein bands were visualized in the Bio-Rad ChemiDoc XRS system (Berkeley) using the HRP substrate chemiluminescence reagent (Millipore, United States).

### Cell Colony Formation

The colony formation assay was applied to analyze the cell survival. After 24 h of transfection, cells were re-inoculated in 6-well plates at a 1 × 10^3^/well density. Then the cells were incubated at 37°C with 5% CO_2_ for about 15 days. The cells in 6-well plates were fixed with methanol for 20 min and stained with crystal violet for 15 min. Colonies were examined and calculated using ImageJ software (Schneider et al., 2012).

### Measurement of Reactive Oxygen Species (ROS)

We used the peroxide-sensitive fluorescent probe DCFDA/H2DCFDA to detect the cellular ROS level. Cells were inoculated in six-well plates and treated with si-NC, si-FANCD2-1, si-FANCD2-2, and TMZ. The level of ROS was determined by a flow cytometer.

### Statistical Analysis

SPSS 19.0 software was applied to perform statistical analysis. A *p*-value of less than 0.05 is statistically significant. All experiments in this study were conducted at least three times with mean ± standard deviations (SD). The student’s *t*-test was adopted to analyze the expression, ROS levels, and cell survival rates. The prognosis for GBM was analyzed with Kaplan-Meier analysis. Wilcoxon rank sum test or Kruskal–Wallis rank test was utilized to evaluate the relation between FANCD2 expression and clinicopathological variables. Furthermore, correlations between genes and immune cells were analyzed using Spearman’s correlation coefficient.

## Results

### Identification of Differentially Expressed FRGs in GBM

**FIGURE 1:**
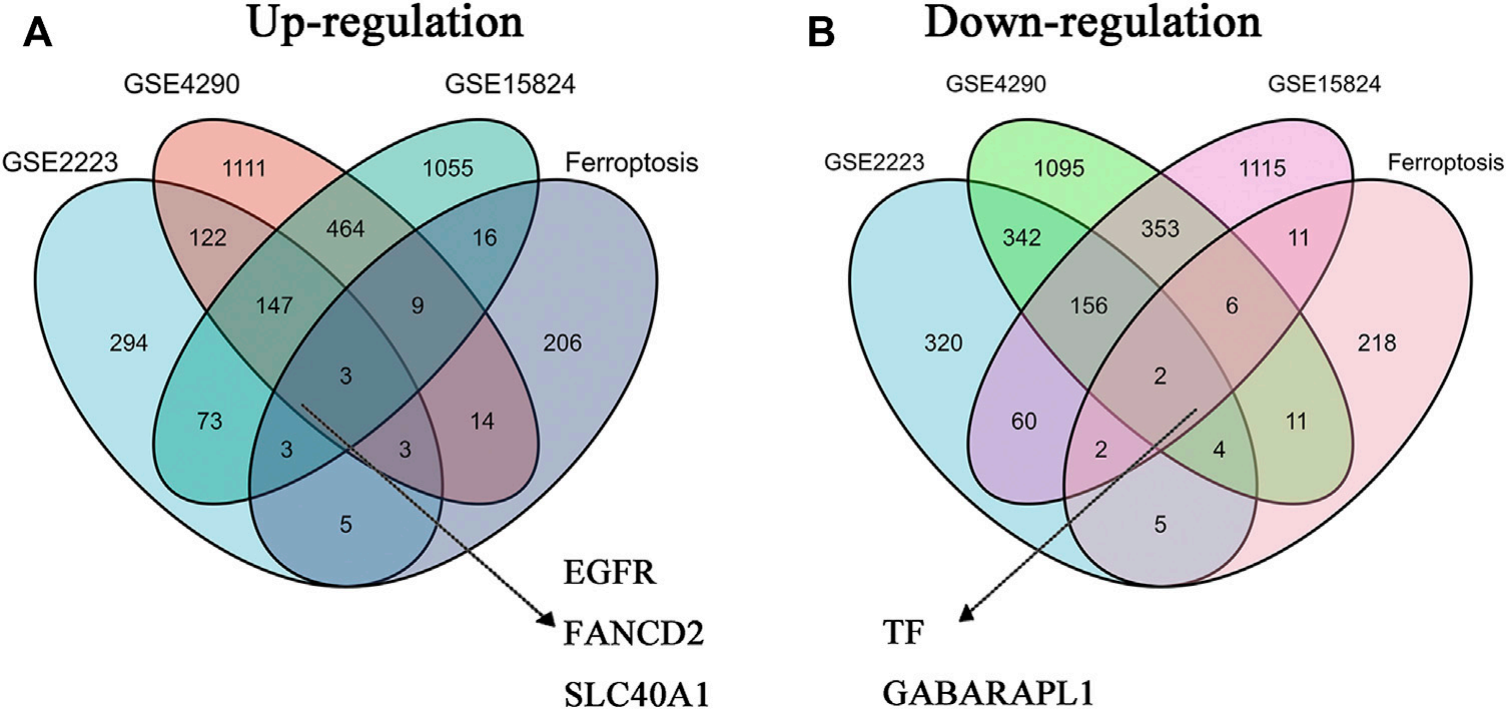
The co-differentially expressed FRGs in three GBM datasets **(A)** Three up-regulated FRGs (EGFR, FANCD2, and SLC40A1) in GBM datasets **(B)** Two downregulated FRGs (TF and GABARAPL1) in GBM datasets.

The co-DEGs in GBM were identified from three GEO datasets (GSE2223, GSE4290, and GSE15824). As shown in Table 1, there are 650 up-regulated genes and 891 down-regulated genes identified in GSE2223, 1873 up-regulated genes and 1969 down-regulated genes identified in GSE4290, and 1770 up-regulated genes and 1708 down-regulated genes identified in GSE15824. The FRGs in GBM were downloaded from the FerrDb database. Additionally, we preliminary identified three up-regulated FRGs (FANCD2, EGFR, and SLC40A1) and two down-regulated FRGs (GABARAPL1 and TF) in GBM tissues through the Venn analysis (Figure 1A,B).

### High Expression Level of FANCD2 Exhibits Poor Prognosis in GBM Patients

**FIGURE 2:**
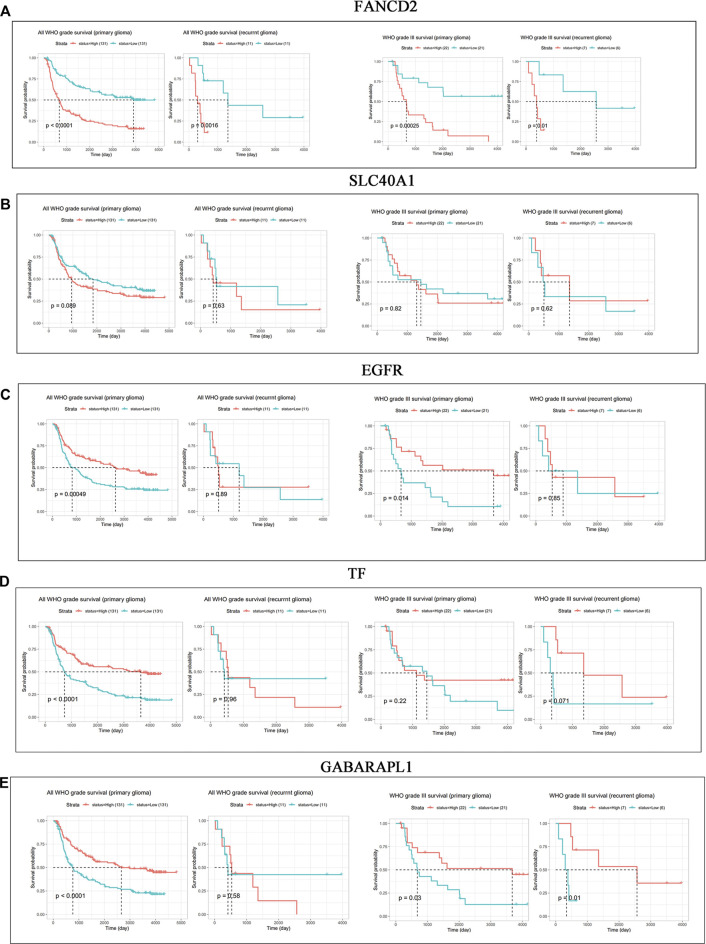
(Continued). Prognostic values of FANCD2, SLC40A1, EGFR, TF, and GABARAPL1 in GBM patients **(A–E)** CGGA database indicated the prognostic values of FANCD2, SLC40A1, EGFR, TF, and GABARAPL1 in patients with primary and recurrent glioma.

The roles of FANCD2, EGFR, SLC40A1, GABARAPL1, and TF in the patients’ prognosis were analyzed based on the CGGA database. In the mRNA_array_301 dataset, patients with high FANCD2 conferred a poor OS in primary glioma of all WHO grades (HR > 1, *p* < 0.0001), recurrent glioma of all WHO grades (HR > 1, *p* = 0.0016), primary glioma of WHO grade III (HR > 1, *p* = 0.00025), and recurrent glioma of WHO grade III (HR > 1, *p* = 0.01) (Figure 2A). However, Figure 2B–E show no significant correlation between the expression of EGFR, SLC40A1, GABARAPL1 or TF and prognosis in recurrent GBM patients (*p* > 0.05). The above results indicated that high expression of FANCD2 may play a key role in the tumorigenesis and therapeutic responses in GBM patients.

## High Expression Level of FANCD2 in GBM Correlates With the Clinicopathologic Features

**FIGURE 3:**
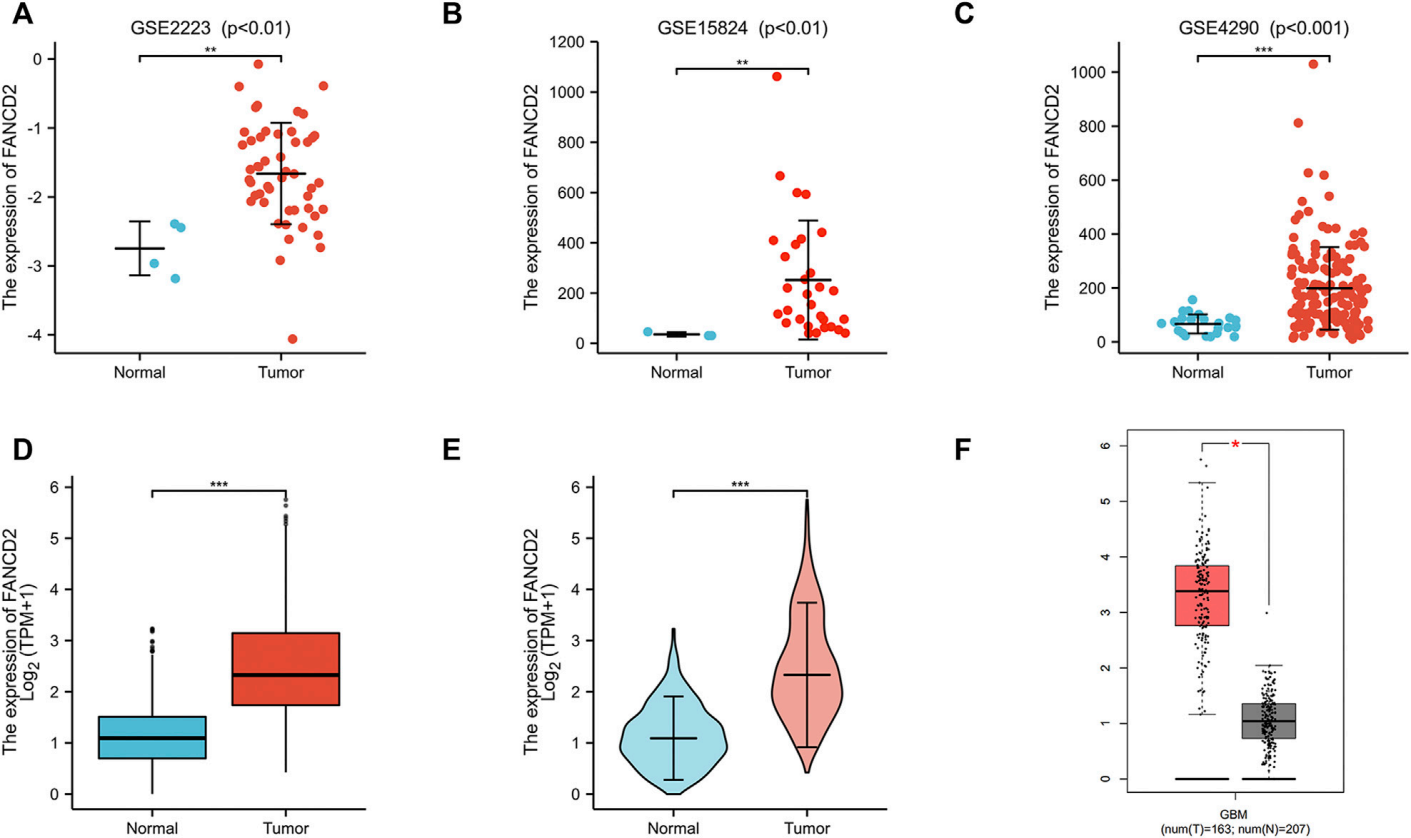
The expression levels of FANCD2 in GBM tissues **(A–C)** Compared with normal brain tissues, FANCD2 expression was significantly increased in GBM tissues from three GEO datasets **(D–E)** The expression level of FANCD2 was up-regulated in the TCGA-GBMLGG cohort **(F)** GEPIA database indicated that FANCD2 expression was dramatically up-regulated in GBM.

Compared with normal brain tissues, FANCD2 expression was significantly up-regulated in GBM tissues in the three GEO datasets (Figure 3A–C). The FANCD2 expression was also up-regulated in the TCGA_GTEx-GBMLGG dataset (Figure 3D,E). Furthermore, the differential expression of FANCD2 between GBM and its corresponding non-cancer tissues can be confirmed by GEPIA (Tang et al., 2017) (Figure 3F). Then, the relationships between FANCD2 expression level and patients’ clinical parameters were analyzed by exploring the TCGA database in the Xiantao bioinformatics toolbox (https://www.xiantao.love/products). Table 2 shows that FANCD2 expression is significantly correlated to the WHO grade (*p* < 0.001), IDH status (*p* < 0.001), and 1p/19q codeletion (*p* < 0.001).

**TABLE 2:** Association between the patients’ clinical characteristics and FANCD2 expression levels in GBM patients.

Characteristics	Low Expression of FANCD2	High Expression of FANCD2	*p* Values
N	348	348	—
WHO grade	—	—	<0.001
G2	180 (28.3%)	44 (6.9%)	—
G3	113 (17.8%)	130 (20.5%)	—
G4	19 (3%)	149 (23.5%)	—
IDH status	—	—	<0.001
WT	54 (7.9%)	192 (28%)	—
Mut	291 (42.4%)	149 (21.7%)	—
1p/19q codeletion	—	—	<0.001
codel	119 (17.3%)	52 (7.5%)	—
non-codel	228 (33.1%)	290 (42.1%)	—
Age	—	—	—
—	40 (32, 52)	52 (37.75, 62.25)	<0.001

### Knockdown of FANCD2 Promote Ferroptosis and TMZ-Sensitivity in GBM Cells

**FIGURE 4:**
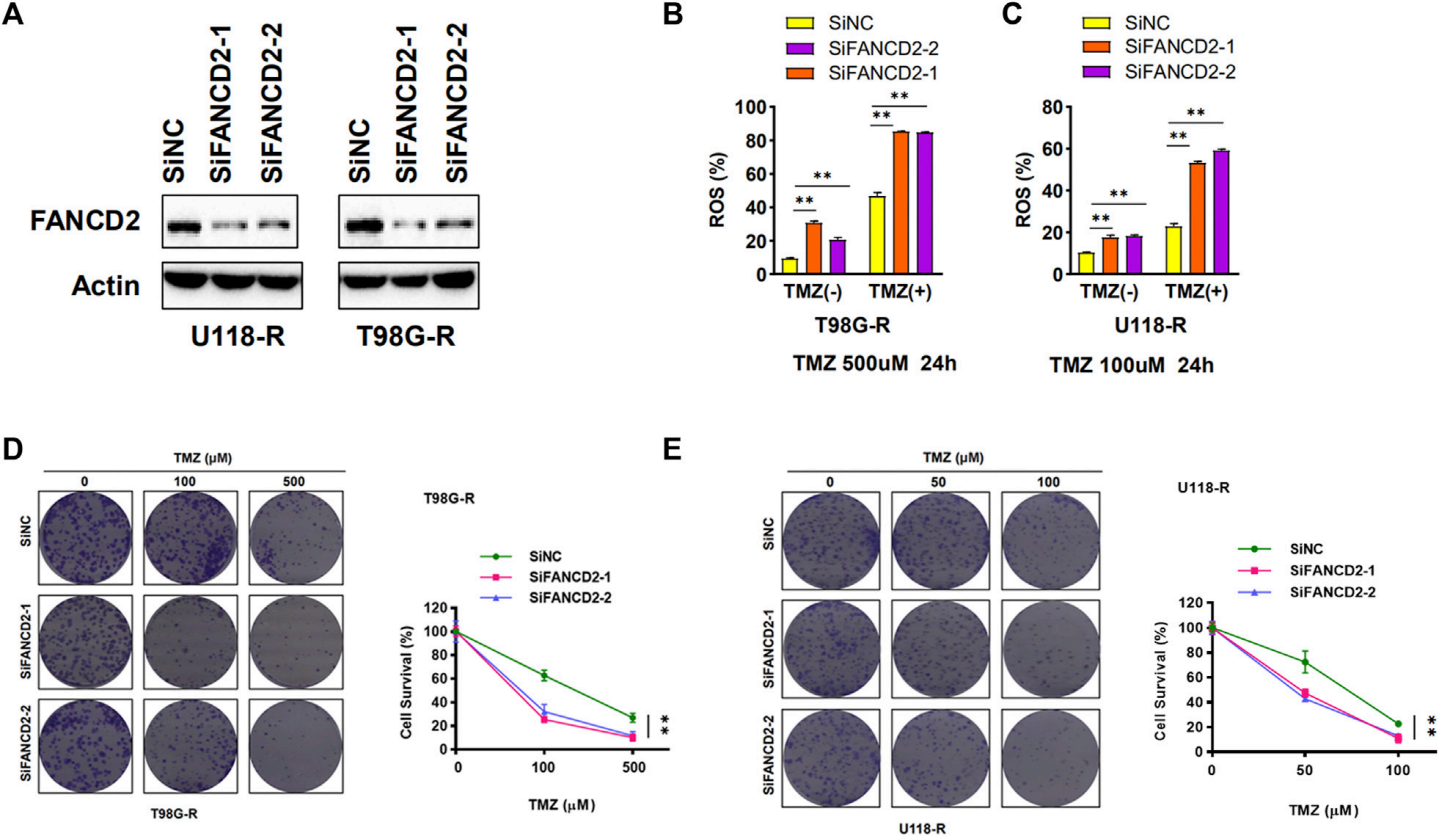
FANCD2 knockdown sensitized GBM cells to TMZ and improved cellular ROS levels **(A)** Two GBM cells (T98G-R and U118-R) were transiently transfected with the FANCD2 siRNA. Cell lysates were then blotted with the indicated antibodies **(B–C)** Cellular ROS levels were up-regulated in FANCD2 knockdown cells **(D–E)** FANCD2 knockdown significantly inhibited the cell survival induced by the TMZ treatment. **p < 0.01.

Two TMZ-resistant GBM cell lines (T98G-R and U118-R) were used to explore the function of FANCD2 on TMZ resistance. siRNA mediated knockdown strategy was applied to inhibit the expression of FANCD2 in TMZ-resistant cells T98G-R and U118-R (Figure 4A). As a characteristic associated with ferroptosis, ROS accumulation was detected to investigate the roles of FANCD1 knockdown on ferroptosis regulation (Chen et al., 2020). The knockdown of FANCD2 could induce TMZ-induced ROS in T98G-R and U118-R cells (Figure 4B,C) and significantly inhibit the cell survival rate induced by the TMZ treatment (Figure 4D,E). These results demonstrate that high expression levels of FANCD2 may promote the TMZ-resistance by preventing ferroptosis in GBM cells.

### FANCD2 Co-Expression Analysis in GBM

**FIGURE 5:**
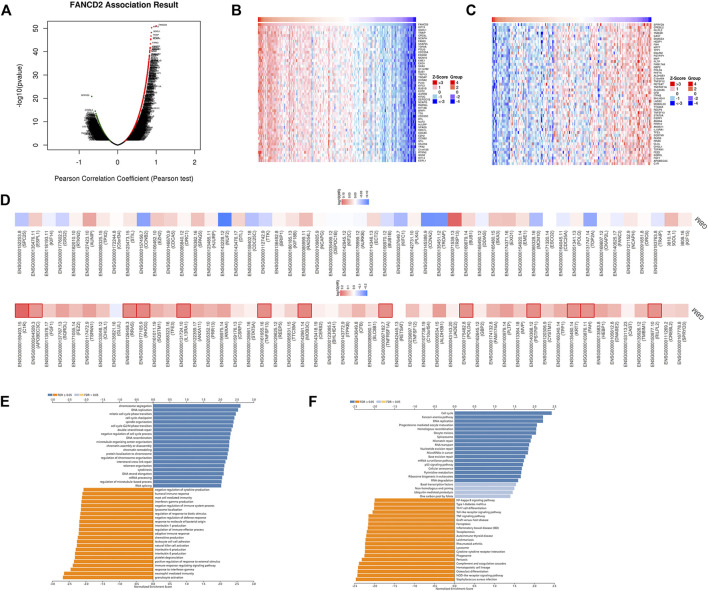
The co-expression genes of FANCD2 in GBM **(A)** LinkedOmics database indicated the significantly correlated genes with FANCD2 in GBM **(B–C)** Heatmaps indicated the top 50 genes displaying positive and negative associations with FANCD2 in GBM **(D)** Survival heatmaps exhibited the prognostic values of the top 50 genes displaying positive and negative association with FANCD2 in GBM **(E–F)** GO and KEGG annotations of FANCD2 co-expression genes in GBM.

To better understand the biological significance of FANCD2 in GBM progression, the co-expression pattern of FNACD2 in the TCGA-GBM cohort was explored using LinkedOmics analysis. As shown in Figure 5A, 4534 genes (red dots) are positively correlated with FANCD2, and 4459 genes (green dots) are negatively correlated with FANCD2. Figure 5B,C show the heatmaps of the top 50 genes positively-related and negatively-related with FANCD2, respectively. In addition, we investigated the prognostic values of these co-expressed genes. As shown in Figure 5D, 54% (27 out of 50) positively-related genes (red boxes) have unfavorable hazard ratios, and 2% (1 of 50) negatively-related genes have protective hazard ratios, indicating that the co-expressed genes have attractive prognostic values for the GBM patients. The co-expressed genes of FANCD2 have been found to participate in several cancer-associated signaling pathways, such as signaling pathways of chromosome segregation, DNA replication, and cell cycle phase transition (Figure 5E). The KEGG pathway analysis also suggested that complement and coagulation cascades and the PPAR signaling pathway were the top enriched pathways regulated by FANCD2 co-expressed genes (Figure 5F).

### The Roles of FANCD2 in Tumor Immune Infiltrations

**FIGURE 6:**
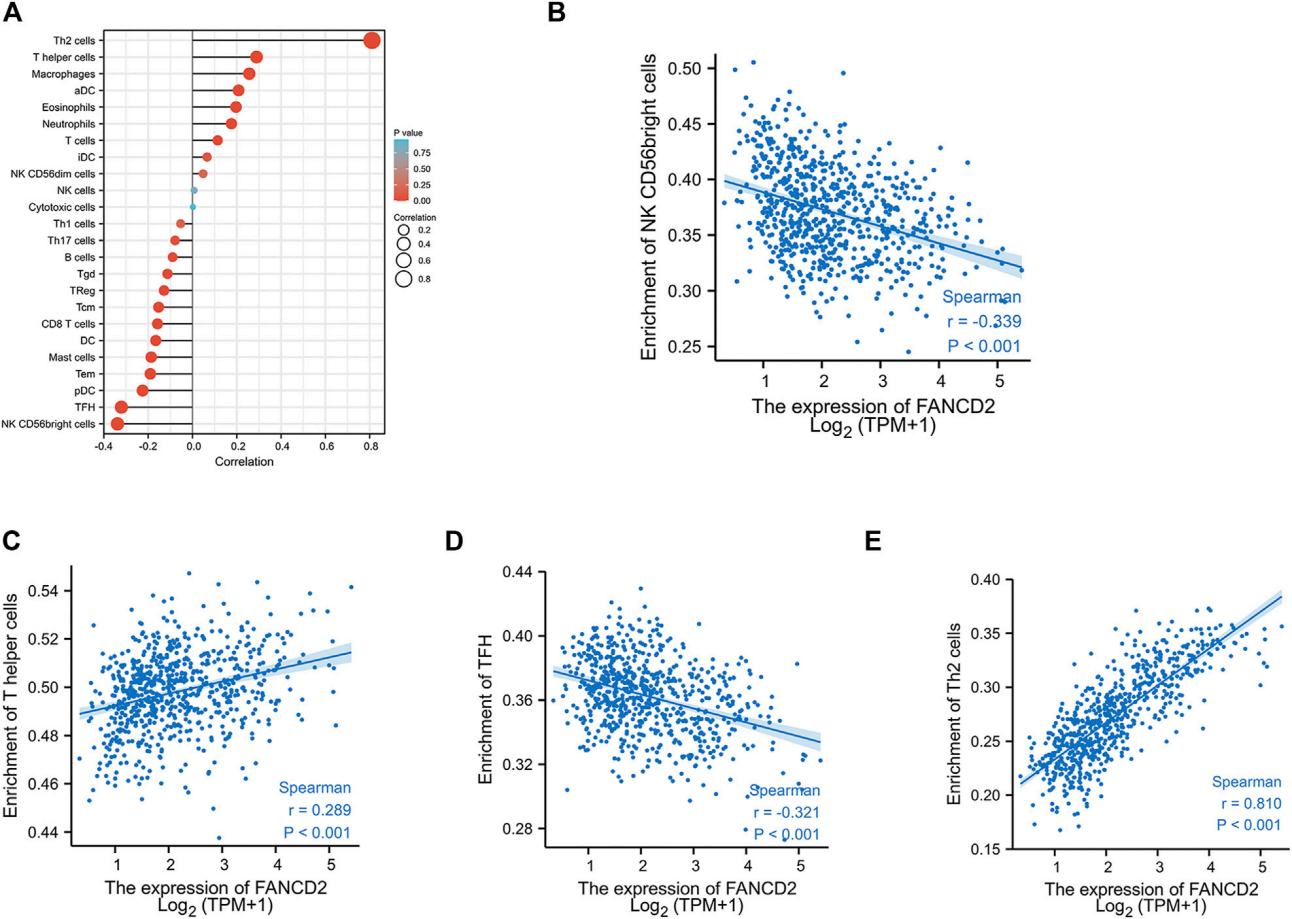
Correlation between FANCD2 expression and tumor-infiltrating lymphocytes in GBM **(A)** The association between FANCD2 expression and 24 types of immune cells in the TCGA database. Absolute values of Spearman r were measured by the size of round dots **(B–E)** The scatterplot showed the association between FANCD2 expression and the abundance of tumor-infiltrating lymphocytes, including Th2, Th, NK-CD56bright, and TFH cells.

**FIGURE 7:**
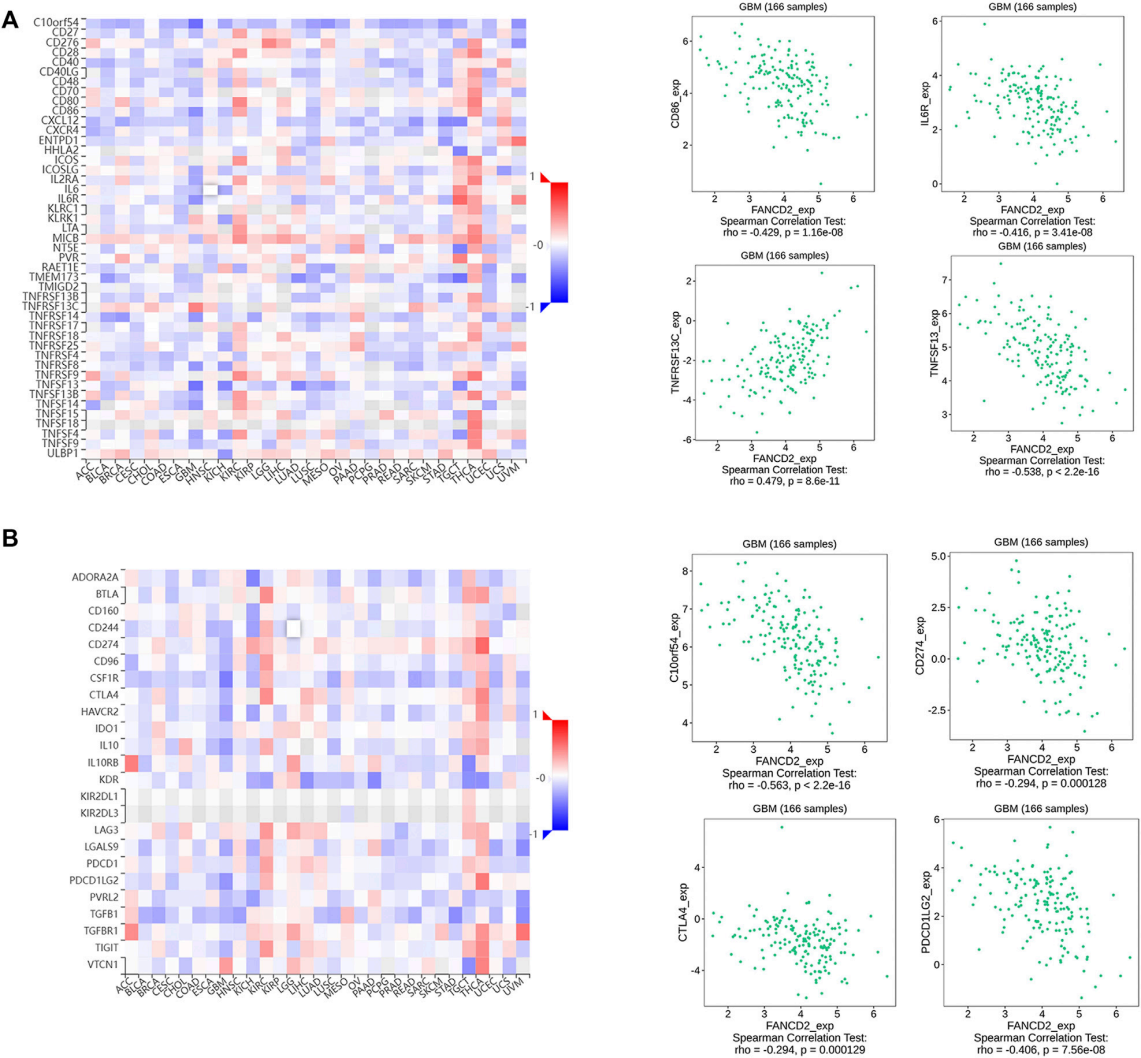
Associations between FANCD2 expression and immunomodulators from the TISIDB database **(A)** The top four immune stimulators (CD86, IL6R, TNFSF13, and TNFSF14) negatively correlated with FANCD2 **(B)** The top four immune inhibitors (C10orf54, PDCD1LG2, CTLA4, and CD274) negatively correlated with FANCD2.

**FIGURE 8:**
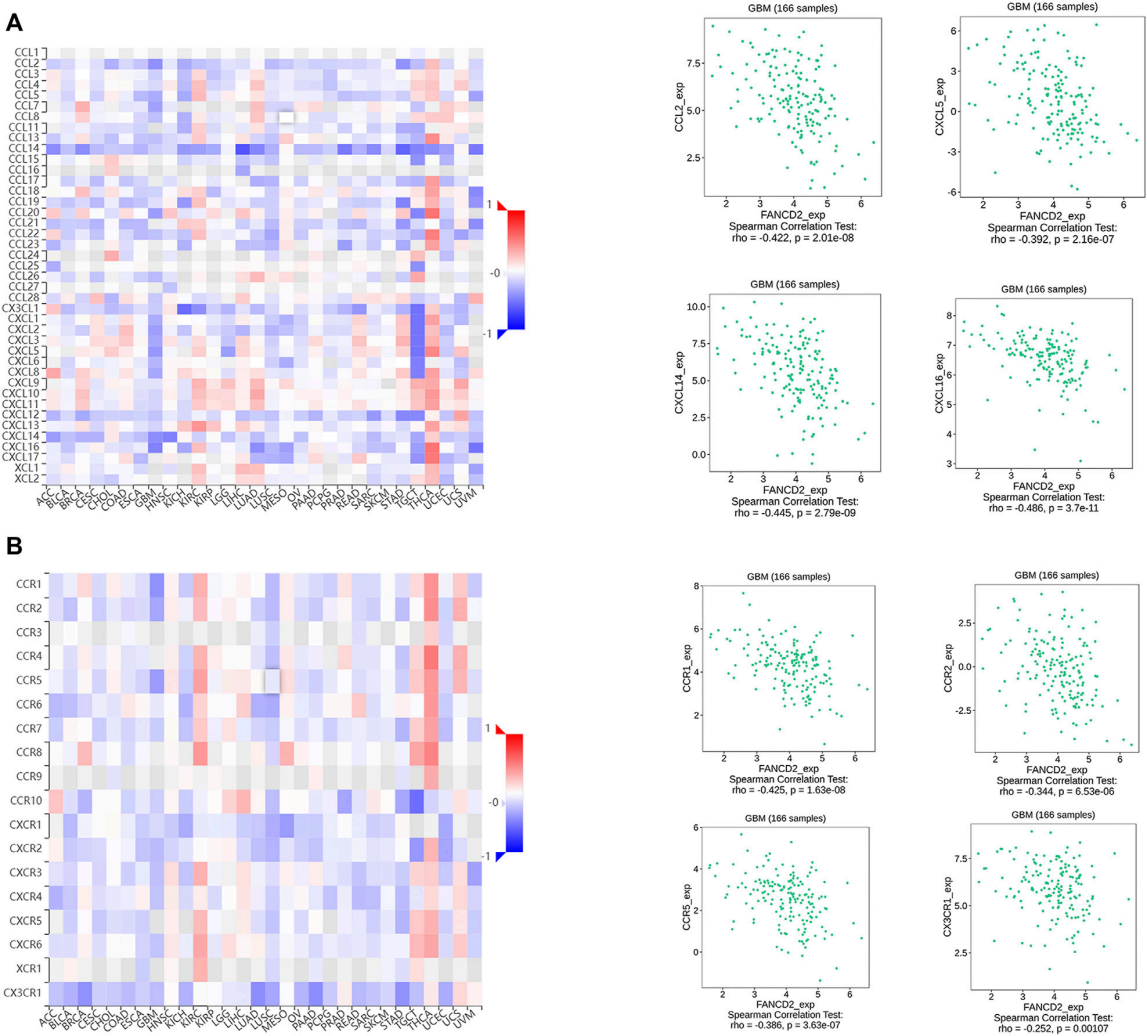
Associations between FANCD2 expression and chemokines-chemokines receptors from the TISIDB database **(A)** The top four chemokines (CCL2, CXCL5, CXCL14, and CXCL16) negatively correlated with FANCD2 **(B)** The top four receptors (CCR1, CCR2, CCR5, and CX3CR1) negatively correlated with FANCD2.

Many reports have demonstrated that ferroptosis may contribute to the immune response in GBM cells (Xu et al., 2022). We analyzed the potential function of FANCD2 in regulating the immune cell infiltration in the TCGA-GBMLGG cohort through Spearman correlation. The results showed that FANCD2 expression was positively correlated with T helper 2 (Th2) and T helper (Th) cells and negatively correlated with NK-CD56bright and follicular helper T cells (TFH) (Figure 6A–E). We further investigated the roles of FANCD2 in immune-associated signatures, such as immune-stimulators, immune-inhibitors, chemokines, and their receptors, in GBM pathogenesis. Figure 7A,B respectively show the top four negatively correlated immune stimulators (CD86, IL6R, TNFSF13, and TNFSF14) and the top four negatively correlated immune inhibitors (C10orf54, PDCD1LG2, CTLA4, and CD274). Figure 8A,B respectively show the top four negatively correlated chemokines (CCL2, CXCL5, CXCL14, and CXCL16) and the top four negatively correlated chemokine receptors (CCR1, CCR2, CCR5, and CX3CR1). These findings suggested that aberrantly expressed FANCD2 might regulate the immune microenvironment in GBM patients.

## Discussion

We explored novel biomarkers involved in ferroptosis and GBM progression by analyzing multiple bioinformatics platforms. Five overlapping genes were found among three GEO datasets and FRGs, including three up-regulated genes (EGFR, FANCD2, and SLC40A1) and two downregulated genes (TF and GABARAPL1). However, only FANCD2 showed potential prognostic values in GBM patients. Additionally, our findings showed that the up-regulated FACND2 was critical to GBM pathological progression and TMZ resistance.

Ferroptosis is a newly discovered regulated cell death characterized by aberrant lipid peroxidation and lethal ROS accumulation. Increasing evidence suggests that ferroptosis is important for inhibiting cancer cells, especially in drug-resistant tumor cells (Tang et al., 2021). A study *in vitro* indicates that quinacrine, a ferroptosis inducer, can impair the autophagic process and increase TMZ sensitivity by triggering ferroptosis in glioblastoma stem-like cells (GSCs) (Buccarelli et al., 2018). Erastin, another ferroptosis inducer, can enhance the sensitivity of TMZ by facilitating ferroptotic cell death in GBM cells (Sehm et al., 2016). In addition, the activity of system Xc-, a key regulatory target of ferroptosis, is markedly induced by TMZ via promoting Nrf2-dependent activation of transcription factor 4 (ATF4) in GBM cells (Chen et al., 2015). Studies mentioned above have demonstrated that ferroptosis can affect the development and treatment of GBM. However, the detailed roles of FRGs in GBM prognosis and TMZ resistance remain unclear. In this study, we firstly identified that ferroptosis suppressor FANCD2 may be a novel therapeutic and prognostic target in GBM. Furthermore, FANCD2, as a DNA damage response regulator, also regulates ferroptosis sensitivity by inhibiting iron accumulation and lipid peroxidation in an autophagy-independent manner ((Sumpter et al., 2016). The FANCD2 has crosstalk with RNA processing and extracellular vesicles to enhance cancer metastasis and drug resistance (Miao et al., 2022). In this study, FANCD2 was up-regulated in GBM and displayed poor prognosis values in patients. Knockdown of FANCD2 attenuated the TMZ resistance by up-regulating ROS level and ferroptosis.

Conventional therapy strategies for GBM include surgical resection, radiotherapy, and TMZ chemotherapy. However, the effects are unsatisfactory. Nowadays, immune checkpoint inhibitors, as the most promising strategies in immunotherapy, play a key role in the field of anti-tumor. It is essential to discover more immune checkpoint targets and develop their roles in anti-tumor research (Ribas and Wolchok, 2018). Immunosuppressive microenvironment and programmed cell death participate in the immunotherapeutic resistance. Liu et al. reported that ferroptosis inducer could combine with immune checkpoint blockade to generate a synergistic therapeutic outcome in GBM murine models (Liu et al., 2022). In this study, we assessed the underlying roles of FANCD2 in the immune response of GBM patients and found that aberrant FANCD2 might regulate multiple immune-associated signatures in GBM pathogenesis, such as immune-stimulators, immune-inhibitors, chemokines, and their receptors.

Furthermore, T helper 1 (Th1) and Th2 cells are the main Th cells involved in anti-tumor immunity. The Th1 exhibits prominent antitumoral activity by activating cytotoxic CD8^+^ T cells and macrophages. The process is linked to tumor regression. The roles of Th2 in cancer immunity are controversial (García de Durango et al., 2021). On the one hand, Th2 cells secrete the inhibitory cytokines to block the differentiation and effector functions of T cells, and promote tumor recurrent (Shimato et al., 2012). On the other hand, Th2-related cytokines can exhibit antitumoral activity by recruiting eosinophils in the tumor environment (Ellis and Riley, 2020). The TFH cells can help B cells with potent antibody responses. In tumor tissues, high levels of TFH cells are often related to favorable outcomes in cancer patients (Baumjohann and Brossart, 2021). Here, we demonstrated that in microenvironments, FANCD2 expression was positively correlated with several TILs, such as Th2, Th, and TFH cells. The FANCD2, associated with various immune regulators, may play a key role in GBM microenvironments and potentially function as an immunotherapeutic target.

Nevertheless, this paper still has some limitations. First, the specific molecular mechanisms of the effects of FANCD2 on TMZ resistance in GBM has not been explored in this paper. Secondly, we need to research the effects of FANCD2 knockdown on survival in animal models. Third, more potential signal molecules containing immune microenvironment involved in GBM progression and TMZ resistance require further investigation. Fourth, ferroptosis-related regulated molecules could be further identified in further study.

## Conclusion

This study has verified that FANCD2, an FRG, is significantly up-regulated in GBM and displays poor prognostic values. The FANCD2 knockdown can sensitize GBM cells to the TMZ treatment, significantly increase cellular ROS levels, and inhibit cell survival rates. Therefore, it is concluded that ferroptosis suppressor FANCD2 may be a promising prognostic and therapeutic target for GBM patients.

## Data Availability

The datasets presented in this study can be found in online repositories. The names of the repository/repositories and accession number(s) can be found in the article/supplementary material.
